# Effects of Low Temperature Stress on Source–Sink Organs in Wheat and Phosphorus Mitigation Strategies

**DOI:** 10.3389/fpls.2022.807844

**Published:** 2022-02-11

**Authors:** Hui Xu, Muhammad A. Hassan, Dongyue Sun, Zhaochen Wu, Gang Jiang, Binbin Liu, Qianqian Ni, Wenkang Yang, Hao Fang, Jincai Li, Xiang Chen

**Affiliations:** ^1^College of Agronomy, Anhui Agricultural University, Hefei, China; ^2^Jiangsu Collaborative Innovation Centre for Modern Crop Production, Nanjing, China

**Keywords:** wheat, low temperature stress, source–sink damage, phosphorus, mitigation strategies

## Abstract

The 21st century presents many challenges to mankind, including climate change, fast growing human population, and serious concerns over food security. Wheat is a leading cereal crop that largely fulfills the global food needs. Low temperature stress accompanied by nutrient-starved soils is badly disrupting the source–sink relationship of wheat, thus causing an acute decline in final yield and deteriorating the grain quality. This review paper aimed to understand how low temperature stress affects wheat source–sink organs (i.e., leaves, roots, and spikes) and how phosphorus application reliefs in alleviating its harmful consequences. Also, we discussed mitigation strategies to enhance wheat capacity to adapt to varying temperature extremes and made rational recommendations based on modern agronomic and breeding approaches. Therefore, this study is likely to establish a solid foundation for improving the tolerance to low temperature stress and to improve its phosphorus utilization efficiency in wheat.

## Introduction

To meet the dietary needs of 10 billion people by 2050, current food productivity will have to rise by 60% (Hickey et al., 2019). Wheat (*Triticum aestivum* L.) is an important cereal crop consumed as a staple food across the globe (Muhammad et al., 2021), which occupies about 220 million hectares of cropland worldwide (Erenstein et al., 2021). As a vital source of plant protein, wheat is easy to be processed into various types of food products, consumed by billions of people, playing an important role in reducing hunger (Subedi et al., 2019; Wojtowicz et al., 2020). According to the latest IPCC report, the global temperature rise is expected to reach or exceed 1.5°C by the end of the 21st century (IPCC, 2021). Global warming increases the instability of the climate system, and extreme low and high temperature events occur frequently (Chen et al., 2019a). Low temperature stress (LTS) causes substantial decline in wheat yield in major wheat-growing regions of the world (e.g., Europe, China, the United States, and Australia; Table 1).

**Table 1:** Effects of LTS on yield and typical events in major wheat producing countries.

Nation	Year	Regions	LTS intensity/Frequency	Yield losses	Typical events	References
United States	1955–2010	Kansas State	41 times	8 bushels /acre in an annual yield loss	LTS damaged nearly half of Kansas wheat, resulting in an average yield reduction of 31% in the spring of 1981.	Holman et al., 2011
Australia	1999–2007	Queensland and northern New South Wales	Extreme LTS reached −6°C or below	10% yield reductions and $73 million economic losses in an average year	18 frosts in the winter of 2000–2002, with the lowest temperature to −8.8°C in 2001, resulted in severe universal damage.	Frederiks et al., 2004, 2008
China	2000–2008	Shandong province	8 times during this 9-year period	More than 10% of the wheat planting area (total area more than 2.92 million ha) was damaged in five growing seasons	Severe frost occurred frequently in (Taishan area) central Shandong province, with the frequency of up to 70%.	Wang et al., 2011; Ji et al., 2017

Global warming conditions accelerate growth and development of wheat, resulting in significant advancement of the low-temperature-sensitive stage of wheat, thus increasing the risks of chilling stress (0 ~ 15°C) or freezing stress (<0°C; Ji et al., 2017; Xue et al., 2019; Liu et al., 2020a). Sub-zero temperatures induce extracellular ice, which severely damages the membranous structures and dehydrates the cell (Roman-Figueroa et al., 2021). It stimulates the dry conditions in roots, leaves, and spikes (Aroca et al., 2012), consequently shortens the plant height, and reduces leaf area and spike size (Valluru et al., 2012). LTS also induces photoinhibition by reducing plant capability to assimilate light energy (Mattila et al., 2020). Generally, the imbalanced source–sink relationship deteriorates the wheat yield and quality by reducing root absorption capacity, declining photosynthetic activity, poor spike differentiation, and delayed grain filling under LTS (Li et al., 2014; Zhang et al., 2019; Kul et al., 2020).

Soil degradation also greatly interferes with wheat productivity (Alewell et al., 2020; Raimondo et al., 2020). Phosphorus (P) is an essential nutrient for wheat growth and development, and about 40% agricultural lands are deficient in P worldwide (Lynch, 2011; Rafiullah et al., 2020). The deficiency of P not only limits the crop growth but also reduces the plant capacity to withstand adverse impacts of LTS (Gong et al., 2020). In soil, P exists in both organic and inorganic form, while plants generally intake the inorganic phosphate (Pi: H_2_PO^4−^ and HPO_4_^2−^) through the adsorption process (Shen et al., 2011). Inadequate P uptake inhibits the development of plant organs, resulting in more prone to the LTS, eventually lower down grain yield (Cong et al., 2020; Oliverio et al., 2020). Many studies described that P application enhanced the drought and salt tolerance in field crops (Bargaz et al., 2016; Karimzadeh et al., 2021). Nevertheless, fewer studies depicted the relationships and interactions between LTS and P application (Cordell and Neset, 2014).

This review article abridged the damaging effects of LTS on wheat source–sink organs and highlighted the prominent breeding and agronomic approaches to enhance the wheat LTS tolerance and PUE by implying recommended crop husbandry practices. Further, various doable measures are proposed to conserve the subsoil phosphate resources, increase the availability of Pi to the roots, and to improve the PUE and LTS tolerance capacities in wheat. This review intended to open-up new research directions for crop stress resistance and mineral resource management.

## Wheat Source–Sink Organs and Risks Associated With LTS

LTS undermines the plant’ ability to smoothly acquire water and nutrients, resulting in chlorotic leaves, stunted growth, disrupted ROS metabolism, floret abortions, and poor seed setting. This section mainly discusses the damaging effects of LTS in wheat from morphological, physiological and molecular perspectives (Tables 2, 3).

**Table 2:** Morphological traits of source-sink organs of wheat, influenced by LTS.

Plant organs	Effect	Growth stage	LTS intensity and duration	LTS induced alterations	References
Root	Root growth inhibited and root contact area reduced	Seedling stage (the first leaf expanded fully)	2 to 5°C (10 h) Control: 25°C	Root relative growth rate ↓	Equiza, 2001
		Seedling stage	10°C (7 d) Control: 20 and 30°C	Root length ↓ Fresh and dry root weight ↓	Buriro et al., 2011
				Root surface area ↓ Branching angle ↓ Lateral roots formation ↓	Nagel et al., 2009; Muhammad et al., 2021
Leaf	Wilting and yellowing, leaf area decreased	Approximately seven leaves stage	−5°C (1,3 d after LTS) Control: 0 d after LTS	Leaf dehydration Wilting and drooping	Han et al., 2013
		Initial seedling stage	4°C (42 d) Control: 4°C	Leaf number ↓ Flag leaf area ↓ Leaf biomass ↓ ^*^Specific leaf area ↓	Valluru et al., 2012
		Seedling stage (three-leaf stage)	2 to 5°C (10 h) Control: 25°C	Leaf area ↓ Leaf thickness ↑	Equiza, 2001
Spike	Spike growth inhibited and grain yield decreased	Six leaf stage, or jointing stage	−3°C (24, 30, and 48 h) Control: 24°C/16°C, day/night	Discoloration and degeneration Spike length ↓	Koo et al., 2008
		Jointing and booting stages	Mean temperature: −6 ~ 2°C (2,4 and 6 d) Control: 6°C	Grain length (L) and width (W) ↓ The grain L/W ratio ↑ Grain appearance quality ↓ ^*^SNPP, GNPP and grain weight ↓ Grain yield and harvest index ↓	Liu et al., 2019a,b

* Specific leaf area, expressed as leaf area/fresh weight or as leaf area/dry weight; SNPP, spike number per plant; GNPP, grain number per plant (Here, ↓ *indicates a decrease and* ↑ *indicates an increase*).

**Table 3:** Physiological and biochemical traits of source-sink organs of wheat, influenced by LTS.

Plant organs	Effect	Growth stage	Low temperature and duration	LTS induced alterations	References
Root	Root absorption ability decreased	Seedling stage (15 d after sowing)	4 ± 1°C (for 14 d); then returned to 20°C	Protein in spring wheat root ↓ Protein in winter wheat root ↑	Karimzadeh, 2000
				Poor plant-nutrient relationships Root activity ↓ Root hydraulic conductivity ↓ ^*^IAA accumulation ↓ ^*^MDA and H_2_O_2_ content ↑ Electrolyte leakage ↑	Aroca et al., 2012; Zörb et al., 2014; Zhu et al., 2015; Hsu and Hsu, 2019
Leaf	Leaf cell structure destroyed, and photosynthesis inhibited	Seedling stage (1 week old)	4°C (0–7 d) Control: 22°C	Photosynthetic electron transport ↓ Photosynthetic apparatus activities ↓ Chlorophyll fluorescence parameter ↓	Venzhik et al., 2011
		Jointing stage	4.8°C (7 d), 14 d recover, then 5.7°C (5 d) Control: 10°C and 14.1°C	Chlorophyll concentration ↓ ^*^Pn and stomatal conductance ↓ Chlorophyll fluorescence parameter ↓ Antioxidant enzyme activities ↑	Li et al., 2014
		Jointing stage	−10 to −3°C (8 h) Control: ambient temperature	The proportion of leaf pigment changed Leaf sponge structure damaged Chlorophyll contents ↓ Photosynthesis levels ↓	Wang et al., 2016b
		The anther connective tissue formation phase	−13 to 0°C (2 h) Control: 4°C	Membrane lipid peroxidation Relative electrolyte leakage rates ↑ Signal transduction proteins ↑	Han et al., 2013
Spike	Flower abortion and grain filling blocked	Booting stage (the young spikes reached the meiosis stage)	4°C (60 h) in 2016 and 5°C/2, 0, −2°C day/night (24 h) in 2017 Control: ambient temperature	Spikelet development inhibited Floret growth delayed ^*^SS and invertase activity ↓ Sucrose content ↑ ^*^SPS activity ↑ ^*^ABA ↑	Zhang et al., 2019
				Fertilization breakdown Starch depletion ^*^Cell wall bound AI activity ↓	Oliver et al., 2010; Thakur et al., 2010
		Jointing and booting stages	Mean temperature: −6 ~ 2°C (2,4 and 6 d) Control: 6°C	Grain nutritional quality ↓ Grain processing quality ↓	Liu et al., 2019b

* IAA, auxins; MDA, malondialdehyde; H_2_O_2_: hydrogen peroxide; Pn, net photosynthetic rate; SS, soluble sugars; SPS, sucrose phosphate synthase; ABA, abscisic acid; AI, acid invertase (Here, ↓ *indicates a decrease and* ↑ *indicates an increase*).

### Root Systems

Roots are considered vital plant organs, which are involved in active nutrient transport from soil to other plant parts (Kim et al., 2020). The root growth is depicted as an ecologically regulated phenomenon (Buriro et al., 2011; Kul et al., 2020). Despite clearly visible detrimental effects of LTS in the aboveground portion of the plant, it also severely disrupts active functioning of root system (Kul et al., 2020).

#### Morphological Alterations

Development patterns and morphological structures of roots are considerably affected by LTS (Shibasaki et al., 2009; Koevoets et al., 2016). LTS restricts the active root growth, particularly upon continuous low temperature exposure, root surface area decreased, branching angle, and contact area narrowed (Nagel et al., 2009). Lateral root formation slower down under LTS (≤10°C), and root volume decreased by 60% than that at 25°C (Nagel et al., 2009). In an experimental investigation, spring wheat and winter wheat were subjected to LTS at 5°C; subsequently, root growth diminished by 60 and 75% as compared to control at 25°C, respectively (Equiza, 2001). Similar results were found in rice, where root length and biomass significantly decreased by 51% under LTS exposure (Hsu and Hsu, 2019; Kul et al., 2020).

#### Imbalanced Biochemical Relations

The active absorption capacity of root systems is vital for maintaining an adequate root–shoot ratio and optimum crop productivity (Equiza, 2001). Sub-optimal conditions disrupt the root water uptake, causing water scarcity in the stem that leads to drought stress; and in the meantime, decreased root absorption capacity and hydraulic conductivity are anticipated with decreased leaf transpiration rate under low temperature and drought conditions (Aroca et al., 2012). Subsequently, drought-induced imbalanced water relations decline the nutrient intake and restrict its transport, ultimately reducing wheat plant growth and development (Nezhadahmadi et al., 2013; Hussain et al., 2018). In an experiment, rice seedlings were subjected to LTS, which led to increased electrolyte leakage, elevated release of hydrogen peroxide and malondialdehyde contents that severely damaged root cells (Hsu and Hsu, 2019). Moreover, LTS also influences the soil’s physio-chemical characteristics that affect the soil microbial activity; later, followed by poor plant–nutrient relations (Yan et al., 2003).

#### Molecular Expressions and Regulation

LTS hinders the biosynthesis of auxins (IAA) by suppressing the gene expression of *ARR1/12* and the gene expression of *PIN1/3/7* that reduces the rootward flow of IAA; subsequently decreased accumulation of IAA in root cells restricts the active cell division of root’s meristematic tissues (Zhu et al., 2015). Thus, poor IAA accumulation is a primary reason for the reduced branching of lateral roots. Sub-optimal conditions give rise to imbalanced osmotic potential, which greatly inhibits active osmoregulation and triggers osmotic and oxidative stress (Sawant et al., 2021). The molecular expression of *TaSnRK2.7* is considered a multifunctional regulatory factor involved in carbohydrate metabolism and reducing osmotic potential in wheat plants under sub-optimal temperature conditions (Zhang et al., 2011). Likewise, other factors, such as phytohormone regulation, expression of *PIP-Aquaporins*, rate of evapotranspiration, and osmotic balance, are thought to be vital for alleviating oxidative stress and active uptake of water and nutrients by roots under low temperature conditions (Aroca et al., 2012; Balliu et al., 2021).

It is briefly concluded that LTS causes a significant reduction in root surface area and lateral branching; subsequently, it interrupts the water and nutrient relations that limits the plant’s capacity to explore more water and mineral resources. There have been few studies that investigated root activity and its molecular expressions under LTS.

### Leaves

Plant leaves are known as food factories because they play an essential role in the process of photosynthesis (PS) and dry matter production. LTS not only instigates root dehydration but leaves as well.

#### Morphological Alterations

Dehydration conditions accompanied by LTS, accelerates the outer membrane destruction, electrolyte leakage in leaf cells, leading to chlorosis, wilting, and eventually necrosis (Dikilitas et al., 2021). Besides, LTS shrinks the leaf area, reduces chlorophyll concentration, alters leaf pigment ratio, and damages the leaf sponge structure (Chinnusamy et al., 2007; Wang et al., 2016b; Zhang et al., 2021b). These morphological changes in leaf area, size, and color are the first visible symptoms of LTS induced damage, and the degree of damage is determined by intensity and duration of LTS (Valluru et al., 2012; Keramidas et al., 2020). In an experimental investigation, wheat plants are exposed to −5°C for 3 d, leaf wilting starts after 1 d and are wholly drooped after 3 d (Han et al., 2013). Fuller et al. (2007) documented that wheat exposure to −3°C for 2 h directly damages the flag leaves, and the damage magnitude intensifies if the temperature drops to −5°C. It is reported that the leaf area index of winter wheat reduced by 43.8% on exposure to below 0°C for 24 h duration, as compared to control (mean temperature: 11°C) treatment (Liu et al., 2019a).

#### Imbalanced Physiological and Biochemical Relations

A plant cell under cold stress experiences severe physiological and biochemical disturbances, which exhibits in leaf chlorosis, wilting, and even necrosis (Ruelland and Zachowski, 2010; Muhammad et al., 2021). The primary sources of wheat grain production are PS and biomass accumulations; these physiological processes are highly vulnerable to LTS (Sharma et al., 2019; Zhang et al., 2020). LTS adversely affects the chloroplast development, chlorophyll biosynthesis, electron transport chain, photophosphorylation, Rubisco efficiency, and carbohydrates transport, resulting in a declining rate of PS and biomass accumulation (Venzhik et al., 2011; Li et al., 2014). Phosphate cycling and photophosphorylation are restricted because LTS inhibits starch and sucrose biosynthesis, resulting in feedback-limited PS (Savitch et al., 2010). Xu et al. (2013) revealed that excessive soluble carbohydrate accumulation inhibits PS and photosynthetic electron transport efficiency by downregulating the level of photosynthetic carbon reduction cycle enzymes. A study has documented an 18% decrease in PS when wheat seedlings were exposed to a low temperature (4°C) in a controlled chamber for 7 d (Cvetkovic et al., 2017). Frost burning of flag leaves resulted in complete restriction of photosynthetic activity (Nevyl and Battaglia, 2021). In cold-sensitive cultivars, photosynthetic activity is more susceptible to cold stress than in cold-tolerant cultivars (Yamori et al., 2009).

ROS is an important indicator for plants to respond to LTS, and its dynamic balance maintains the cell stability and normal plant growth (Sharma et al., 2012). LTS instigates the ROS imbalance by reducing molecular oxygen and producing ROS, and this imbalance is extremely damaging to metabolic processes (Muhammad et al., 2021). With the increase of low temperature frequency, intensity, and duration, extreme LTS occurred that led to excessive accumulation of ROS and membrane lipid peroxidation and subsequently caused significant damage to the chloroplast structure, photosynthetic apparatus, and accelerated the leaf senescence (Huang et al., 2019; Liu et al., 2020b).

#### Molecular Expressions and Regulation

Environmental variations enforced crop plants to evolve diverse adaptation approaches (Chong et al., 2021). The accumulation of soluble sugars, several amino acids, and the expression of antifreeze proteins (PS and electron transfer related protein) in wheat leaves contribute in maintenance of cell turgidity through osmotic balance and the increased capacity of frost tolerance (Markovskaya et al., 2010; Yang et al., 2011; Xu et al., 2013). Many transcriptional studies of *Arabidopsis thaliana* and wheat shows that LTS significantly inhibits the expression of PS-related genes (Gulick et al., 2005; Gan et al., 2019); in which C-repeat/dehydration-responsive element binding transcription factors mediates low temperature signal transduction pathway (Arshad et al., 2016; Jiang et al., 2020). Abscisic acid (ABA) regulation genes, such as *TaMYB33,* are involved in the production of antioxidants, which are beneficial to ROS scavenging, proline accumulation, and osmotic balance (Qin et al., 2012). Likewise, in another experimental study, *TaSAG3* and *TaSAG5* have expressed naturally in wheat leaf senescence; followed by low temperature treatment exhibited stable expressions for *TaSAG5* and highly instable expressions for *TaSAG3* at a low level (Zhao et al., 2012). At this point, ROS transforms from second messengers to cell killers. The mechanism of how abiotic stress affects plant redox states is not clear yet, needs more experimental studies to explore (Huang et al., 2019).

In brief, LTS has drastic impacts on leaf morphology and interior cell structure. Excessive production of ROS disrupts the photosynthetic activity by hindering active electron transfer and accelerated leaf senescence. The decrease of leaf area and net photosynthetic rate resulted in the decrease of assimilates and other photosynthetic products.

### Spikes

LTS hinders the wheat growth at all stages, from seedling to maturity, but the intensity of the damage varies (Muhammad et al., 2021). Many experimental studies established that wheat is more sensitive to LTS at the reproductive stage than vegetative, particularly its susceptibility higher from heading to anthesis, that leads to significant yield losses (Barlow et al., 2015; Zhang et al., 2021d). The top spikelets are considered more sensitive to LTS, followed by the basal and central spikelets (Liu et al., 2020a). The negative effects of LTS not only depends on intensity and duration of LTS but also on crop cultivars and soil fertility (Soares et al., 2019).

#### Morpho-Physiological Impairments and Molecular Expressions Associated With LTS

The negative impacts of LTS on spike development are well documented; most crucial growth period, associated with LTS, begins after pollen development enters meiosis phase prior to anthesis (Slafer et al., 2001). LTS results in short statured spikes, reduced number of spikelets, pollination failure, poor grain filling, and inadequate seed setting (Liu et al., 2019a). Wheat plant’s upper spikelets turns pale yellow or partially dies on exposure to LTS at booting stage (Kodra et al., 2011; Sanghera et al., 2011). Dehydration accompanied by LTS results in abnormal pollen and ovule development, leading to pollen inactivation, floret sterility, and eventually cause abortion (Ali and Malik, 2021). Denatured spikelets, reduced assimilate transport, decreased dry matter accumulation, and poor grain yield are all consequences of LTS (Whaley et al., 2004).

LTS interrupts the balance of source–sink relationship by limiting key physiological process of sucrose accumulation to wheat spikes (Zhang et al., 2019). LTS induced excessive ROS production limits the photosynthetic activity as well as respiration activity that subsequently leads to oxidative damage to sink organs (i.e., spike; anther’s desiccation). In previous studies, LTS induced short statured spikes were attributed to the overexpression of *miRNA156* (Liu et al., 2017). Furthermore, LTS induced ABA pathway affects the sucrose metabolism and related gene expressions in spikelets, subsequently delaying floret and spikelet development (Zhang et al., 2019). The similar trends were found in rice (*Oryza sativa* L.) in which acid invertase (AI) activity was inhibited under LTS, and the quantity of sucrose transportation to the pollen grains and tapetum severely decreased, thus leading to pollen abortion (Oliver et al., 2007). However, it is not clear whether the frost damage in wheat at anthesis is due to the lack of acclimation expressions or the insufficient environmental stimulus to upregulate the acclimation genes (Fuller et al., 2007). Recently, a research finding revealed that wheat spike development under cold conditions in early spring was accelerated due to increased ABA contents and the upregulation of encoding *PP2Cs*, *SnRK2s*, and *bZIP* transcription factors (Yu et al., 2021).

#### Grain Yield and Quality Losses Associated With LTS

Worldwide, researchers are working hard to improve the wheat’s yield, quality, and adaptability to various environmental conditions (Osman et al., 2016; Fleitas et al., 2020). The adverse effects of LTS on yield of wheat plants before heading stage can be compensated by the development of newly formed tillers (Frederiks et al., 2012), whereas yield damage caused by late spring coldness to wheat tillers and newly formed spikes cannot be rectified. This is due to reduction in grain number per plant (GNPP), spike number per plant (SNPP), and grains number per spike (GNPP = SNPP × GNPS; Liu et al., 2020a). The yield of two cultivars (with varying temperature tolerance capacity) decreased by 4.6 and 13.9% under LTS (2°C/0°C, day/night) at jointing stage for 24 h, respectively; and the damages to tillers and spikes are the main reason for reduction in grain yield per plant (GYPP; Li et al., 2015). The grain yield per plant of spring wheat and semi-winter wheat decreased between 4.6–56.4% and 3.1–44.6% under LTS (the mean temperature in the range of −1 to 3°C) at jointing stage, respectively, while decreased by 13.9–85.2% and 3.2–85.9% under LTS at booting stage, respectively (Liu et al., 2019b).

LTS considerably deteriorates the grain quality. Study has shown that sub-optimal temperature conditions have a detrimental impacts on assimilate accumulation during grain filling, leading to deterioration of grain quality and quantity (Yang and Zhang, 2006). Since LTS inhibited both the absorption and distribution of beneficial nutrient elements, nitrogen (N) compounds and non-structural carbohydrates did not reach spikes (Zhang et al., 2021a). Additionally, the decreased canopy temperature resulted in reduced starch and protein accumulation, followed by deformed grain appearance (grain length and size; Liu et al., 2019b).

In summary, LTS limits the normal spike development, which causes severe reduction in grain yield and quality. The effects of LTS on yield loss at early growth stages (vegetative: emergence, tillering, etc.) are not as detrimental as those of later growth stages (reproductive: booting, grain filling, etc.). The impact of LTS on wheat spikes varies with varying temperature intensity and duration, growth phases, and cultivars.

## LTS Risks in Wheat Under P-Starved Soil Conditions and Role of P

It is evident from various experimental investigations that optimum mineral nutrition is vital in combating abiotic stresses, that is, LTS, salinity stress, and drought stress (Waraich et al., 2011; Bouain et al., 2019). P as an essential plant nutrient, not only take part in various metabolic activities (i.e., PS, carbohydrates accumulation, assimilate transportation and distribution, and electron transport), but also vital in strengthening the plant’s adaptability to the external environmental factors *via* taking part in various biological pathways (i.e., signal transduction, energy kinetics, enzymatic catalysis; Soetan et al., 2009). In general, P absorption is primarily affected by temperature change; therefore, in cold weather conditions, P application could significantly increase wheat growth and development. In this section, we discussed how deficient soil conditions coupled with LTS adversely affect the active growth of source–sink organs in wheat.

### Soil P Starvation and Effects on Wheat Plant

Fertilizer consumptions have been steadily increasing since the green revolution, but wheat productivity has remained stagnant in last decade. Since use of different fertilizer application methods and varying soil nutrients supplying capacities, there were large variations noted in wheat nutrient use efficiencies (Jat and Gerard, 2014). In modern-day crop cultivation, P is an integral part of plant metabolism, ranked second essential nutrient after N (Nedelciu et al., 2020). It is expected that global P demand will exceed the supply in 2045 (Nedelciu et al., 2020). Despite the high input of chemical fertilizer, global P shortage is intensified by soil erosion (Alewell et al., 2020). Nowadays, about 50% of agricultural lands across the world are facing P deficiency, which is becoming a serious threat to crop production (Lynch, 2011). To meet the food demands of increasing population, it requires effective measures to prevent fertile agricultural lands from P starvation.

It is known that many abiotic factors are simultaneously influencing the depletion of soil mineral resources, and there is already a shortage of available phosphate in soil (Lynch, 2011). P is relatively easy to be fixed in the soil and becomes immovable. The P-deficient soil accelerates the increase in sucrose transport through phloem toward roots, where Pi transporters cause outward efflux and cause secretions of organic phosphatic acids (Hammond and White, 2007). The P deficiency symptoms are clearly visible in wheat plants: short stature, slender stems/peduncles, yellowish spots on leaves, delayed flowering, poor grain filling, and late maturity (Gross et al., 2021). It further hinders the primary root growth (Linkohr et al., 2002). Moreover, P deficiency directly influences the normal functioning of biochemical and physiological activities, that is, disrupted cell division and protein nucleation (Linkohr et al., 2002; Péret et al., 2011).

Nevertheless, plants have tendency to improve the uptake and utilization of P by changing root system architecture (RSA) and inducing the expression of P starvation response genes (Baek et al., 2017; Cho et al., 2021). It is verified from research investigations that protein expression of *TaPHT2; 1* is a significant factor in P signaling, and its upregulated expression under P-deficient conditions is beneficial in increasing Pi concentration in chloroplasts, enhancing photosynthetic capacity, and P accumulation in wheat plants (Guo et al., 2013; Aziz et al., 2014; De et al., 2019).

### Role of P in Alleviating the Risks of LTS to Wheat

**Figure 1:**
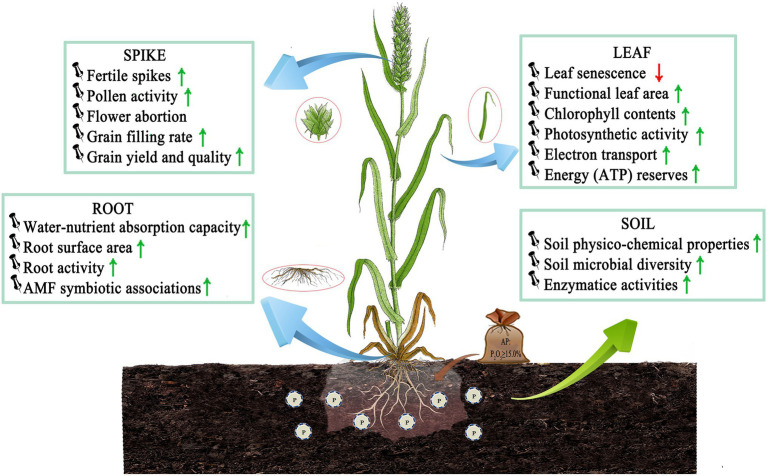
LTS tolerance mechanism by optimizing P application in wheat. Optimizing P application improves soil–plant nutrient relationships, maintains source-sink balance and membrane stability, relieves dehydration and oxidative stress, and enhances crop stress resistance and productivity in wheat. And root surface area and root activity increase in roots; functional leaf area, PS and photoproduct increase in leaves; flower abortion decreases, but pollen activity, grain yield and quality increase in spikes by P application, respectively. AMF: arbuscular mycorrhizal fungi, ATP: adenosine triphosphate [Here, ↓ indicates a decrease and ↑ indicates an increase/improvement].

The optimal dose of P is needed in wheat plants to enhance primary root growth, improve water–nutrient relations and osmotic stress tolerance, and enhance photosynthetic activity facilitated by increased leaf chlorophyll content and dry matter accumulation (Figure 1). These traits are critical in defining the production quantity and quality in wheat. In this section, we briefly discussed the role of P in low temperature resistance in combination with the source–sink pools of wheat.

#### Root Systems

Many studies have focused on the aboveground part and ignored the belowground part (roots) of plants, but root systems play a vital role in mitigating the adverse effects of LTS (Ambroise et al., 2020). Beneficial microorganisms and mineral nutrients are helpful to improve the cold tolerance of wheat roots (Zhou et al., 2021). Although P is relatively easy to be fixed in the soil and becomes immovable, but soil microbial activities are critical in converting insoluble phosphates into soluble phosphates, phosphate solubilizing bacteria (PSB) play a key role in this process (Tabassum et al., 2017; Chatterjee et al., 2021). The P fertilization is recommended to alleviate the negative impacts of LTS and rectify the deficiency symptoms in wheat. It also enhances the belowground microbial activity which is mutually beneficial for healthy root growth and development. Increased microbial activity excites soil temperature to a certain extent that contributes in alleviating the adverse impacts of LTS.

Further, P plays an important role in regulating root system responses to drought stress (Jin et al., 2015; Attarzadeh et al., 2020), because P application prevents crop plants from oxidative stress and improve chloroplast structure, while excessive P application trigger ROS toxicity (Shibli et al., 2006; Noor et al., 2021). Combined with N and potassium (K), P application promotes the secondary root growth, proliferation of growing root tips, and root hair differentiation; subsequently root surface area increased that supports in active nutrient–water uptake (Gahoonia et al., 1999).

An efficient RSA is vital for adequate utilization of mineral and water resources under LTS. Dynamically developed RSA favors the active uptake of available phosphate and other mineral nutrients for the optimal growth of aboveground plant organs (Shen et al., 2011; Dijkstra et al., 2016). Adequate P supply accelerates root growth and its spread in deeper soil layers (Jin et al., 2015). It is reported that beneficial relationship between arbuscular mycorrhizal fungi (AMF) promote P uptake and utilization. AMF is vital in stabilizing soil aggregates and regulating the non-nutritional functioning in field crops that enhance the LTS tolerance by preventing membrane lipid peroxidation (Zhu et al., 2010; Latef and He, 2011; Gerz et al., 2018). Similarly, strigolactones (SLs) secretions from root play a significant role in active N and P uptake by establishing the symbiotic relationship between plants and soil microbes, and it is also crucial in tolerating various biotic and abiotic stresses (Halouzka et al., 2020; Jamil et al., 2020). According to De et al. (2019), two wheat cultivars with different PUEs on fertilization exhibited 60 and 80% increases, respectively, while their PUEs varies by 17% upon no fertilization; this increase in PUE is due to SLs-induced regulation of *PHO2* activity.

In brief, application of P not only improves the soil P deficiency, but also improves the RSA of wheat that is key factor in active nutrient uptake and mitigating biotic and abiotic stresses. In addition, AMF, PSB, and SLs promote the active P absorption from soil.

#### Leaves

Leaves are considered as primary source organs of the plant. Several environmental factors negatively influence leaf functioning and hinder optimal crop growth (Gong et al., 2020). PS is the key physiological process that mainly takes place in the leaf. P fertilization is essential for the development of healthy wheat leaf and maintains the normal PS process (Sharma et al., 2019). LTS repercussions (i.e., membrane impairment, imbalanced osmoregulation, and excessive ROS production) substantially reduced the rate of PS. In this aspect, P fertilization is a handy tactic in alleviating the negative impacts of LTS.

The optimal dose of P is not only helpful in sustaining membrane stability and increasing leaf surface area, but also enhances the leaf chlorophyll and carotenoid contents; subsequently, it increases the rate of PS, enhances the dry matter production, improves the stomatal conductivity and improved the plant–water relations (Waraich et al., 2011; Shirmohammadi et al., 2020). Optimal allocation of P in plants can prolong and improve the P utilization in PS (Veneklaas et al., 2012). It has been reported that foliar application of plant growth regulators (i.e., SLs, salicylic acid, and ABA) significantly enhanced the activities of antioxidant enzymes, which facilitates in preventing membrane peroxidation under drought and LTS (Sun et al., 2009; Ma et al., 2017; Saleem et al., 2021). Accumulation of fructans in cereal crops upon experiencing low temperature environmental conditions is another preventive approach in maintaining membrane stability (Livingston et al., 2009). In a field experiment, combined P (200 mg P_2_O_5_ kg^−1^ soil) and molybdenum fertilizer at the tillering stage of winter wheat upshots the leaf soluble sugars contents by 9.7% and reduced the malondialdehyde content by 28.4% (Nie et al., 2015). In addition, P application under abiotic stress also increases the uptake and utilization of N, increases the concentration of soluble sugar and Pi in plants, and high Pi availability for carbon assimilation maximizes leaves photosynthetic activity (Jin et al., 2015).

P supply relieved the symptoms of LTS through modulating the activities of antioxidant enzymes and maintaining the regular osmotic homeostasis in leaves. Normal morphology and physiology of wheat leaves can maintain the sustainability of PS, the vital ingredient for plant growth and total biomass production.

#### Spikes

Wheat spikes are known as the primary sink pool of wheat, which accumulates the maximum number of photosynthates. LTS induce deformities in spike shape and appearance and badly influences SNPP, GNPP, spike length, and 1,000-grain weight. These basic agronomic parameters are key components of the final wheat yield (Chen et al., 2019b). P deficiency combined with LTS causes a drastic reduction in the number of spikes and productive tillers (El Mazlouzi et al., 2020a). Application of P under LTS maintains the source–sink balance through promoting photosynthetic activity and improving grain filling rate (Engels et al., 2012; Zangani et al., 2021). The vegetative organs (i.e., leaves, stems, and roots) in wheat used as P reserves, which later was transported to harvest organs (i.e., spikes), after anthesis, to promote grain filling and ripening, as P deposition in the grains was mainly brought through remobilization of internal P sources stored before anthesis (El Mazlouzi et al., 2020b). The transport and accumulation of starch and protein to ear improves wheat quality (Kizilgeci, 2019). This enhanced relocation and accumulation of P from source organs to sink organs supported through Pi transporters, H^+^-ATPase, phospholipids, and carbon-metabolism gene expressions (Aziz et al., 2014). Therefore, it is important to supplement sufficient P at the vegetative growth stage to safeguard optimal spike and grain development and stress tolerance in wheat.

It is described that P fertilization significantly increased the spike length, spike number, grains per spike, and 1,000-grain weight, which directly influenced the wheat yield (Saqib et al., 2015; Arshad et al., 2016). In a field trial of wheat broadcasted phosphate fertilizer at the rate of 90 kg P_2_O_5_ ha^−1^, substantial increase was noted in plant height, spike length, GNPP, 1000-grain weight, P concentration, P uptake, and GYPP by 12.8, 7.7, 13.7, 22.4, 24.0, 85.5, and 60.6%, respectively, as compared to control treatment with no P application (Shafi et al., 2020). Moreover, increased accumulation of SS was also observed in various reproductive organs; augmented sugar contents improved the pollen activity, which significantly prevented the floret abortion and spike damage under temperature stress (Zhang et al., 2017).

Currently, little research is being conducted on the role of P fertilization in sustaining source–sink balance and plant–nutrient relations for LTS. Although its actual mechanism is not fully explored yet, a comprehensive and systematic approach study is required to unleash the role of P in the alleviation of risks anticipated with LTS.

## Strategies to Mitigate the Effects of LTS on Wheat and P Regulation

To strengthen the wheat’s capacity in combating the negative effects of LTS, a number of integrated management approaches are being implemented. It includes modern crop breeding techniques (gene mapping, inducing LTS-tolerant genes, omics, etc.), improved crop husbandry practices (seed enhancements, fertilizer management, adequate sowing techniques, timely sowing irrigation management, etc.), and crop modeling approaches (for optimizing the resources and estimating the risks of temperature variability). In this section, we briefly discussed the above-mentioned crop management strategies with regard to improved P regulation.

### Utilizing Modern Breeding Techniques and Tools for Developing P-Efficient and Temperature Resilient Cultivars

Every year, breeders work hard to create cultivars that are better adapted to changing climates. The ongoing research work on wheat LTS tolerance mechanism needs to be combined with modern breeding techniques (i.e., fast breeding, space breeding, and CRISPR-Cas9) for the development of efficient and temperature resilient crop varieties (Hristov et al., 2007; Zhao et al., 2020; Li et al., 2021). For efficient utilization of P, more research is needed into the genetic and molecular mechanisms related to LTS.

To develop a new variety, traditional breeding techniques take more than 10 years, which are not enough to meet the present problems of climatic fluctuations and nutrient deficient soil conditions (Muhammad et al., 2021). The current complex ecological system and abnormal climatic variations posing a severe threat to normal crop growth that restraining the efficiency of existing crop cultivars for longer duration. Therefore, in future, along with opting genotypic marker-assisted and high flux phenotypic selection techniques (Mickelbart et al., 2015; Younis et al., 2020), employing crop simulation models (e.g., CERES-Wheat and DSAT) for predicting the lifespan of particular cultivars in different climatic zones is quite useful (Koç, 2020).

Genome-wide association studies (GWAS) thought to be pragmatic in identifying the genetic factors controlling the phenotypic variability of PUE in wheat (Bin Safdar et al., 2021). In the meantime, GWAS is useful in understanding the genetic basis of LTS tolerance and in developing LTS-resistant wheat cultivars. GWAS-based research reported that *PHO2* and zinc finger transcription factors are involved in many functional and regulatory processes in plants, such as P homeostasis and ROS metabolism (Devaiah et al., 2007; De et al., 2019). Later, the identified genes sources subjected to the site-specific by CRISPR-Cas9 analysis for promoting the development of the efficient P utilization and improved LTS-resistant wheat varieties (Doudna and Charpentier, 2014). The response of different wheat genotypes to LTS and P nutrient was significantly varies, and so, the effective combination of LTS-tolerant and P-efficient genotypes is good way to develop LTS-resistant cultivars; then later followed by domestication of newly developed P-efficient and LTS-tolerant wheat cultivars (Wang et al., 2016a). Recently, a new wheat-*Thinopyrum intermedium* introgression line with tolerance to P deficiency was identified, which provides, which really a new insight into wheat germplasm resources and aids in developing high P-efficient-stress tolerant cultivars (Zhang et al., 2021c).

### Optimizing P Management and Crop Husbandry Practices

Only breeding high P-efficient cultivars is not enough to counter low temperature repercussions, but it is also crucial to implement optimal crop husbandry practices. According to the sustainable development goals of the United Nations, P management is a crucial element in determining final grain yield (Griggs et al., 2014; Rafiullah et al., 2020). Therefore, it is needed to pay attention to the adequate fertilizer application, appropriate timing, and suitable method of fertilization. During the vegetative stage of wheat, P absorption rate is maximum and healthy source organs are needed to combat the negative impacts of LTS at sensitive reproductive (jointing and booting) stages; therefore, adequate P supply must be ensured before anthesis (Liu et al., 2020a).

#### Improved PUE by Implying Appropriate Method of P Fertilization

There are many P application methods, including broadcasting, top dressing, fertigation, foliar application, and P-amendments of biochar. The efficiency of each application method varies. Foliar application of potassium dihydrogen phosphate effectively combat the LTS at critical jointing and booting stages and enhanced the final wheat yield (Rafiullah et al., 2020). Drip fertigation of phosphate fertilizer allows nutrients to reach the root zone directly and improve the P concentration in plant tissue and PUE (Mikkelsen, 1989). Biochar is an emerging soil amendment approach; biochar as P-amendment significantly increased the source of soil organophosphorus, improved the soil quality, and enhanced the efficiency of plant nutrient uptake (Manolikaki et al., 2016). Biochar amendments enables the mobilization soil P through altering soil pH and supporting AMF in developing symbiotic relationships with plant roots (Shen et al., 2016). Combined applications of organic and inorganic fertilizers play a similar role in ensuring P availability for sustainable crop production (Yadav et al., 2021).

#### Measures to Improve Bioavailability of Phosphorus and LTS Tolerance

Conservation agricultural practices are very handy in improving soil health and ensuring bioavailability of essential nutrients in soil (Yang et al., 2020). In South Asia, farmers have already started implementing conservation farming practices, such as reduced tillage and residue retention (Jat and Gerard, 2014). After paddy harvesting, the incorporation of straw residues is an important tillage practice, termed as straw retention. Straw residues are rich in organic carbon, mineral nutrients, and rich biochar amendment for next wheat crop (Arif et al., 2017). Alternatively, inoculation of PSB and mutually beneficial AMF associations effectively improve the bioavailability of phosphate. Further, it promotes active plant growth and minimize the negative implications of stress by establishing a positive relationship with soil mineral nutrients (Khan et al., 2009; Etesami et al., 2021). It is known that PSB plays a vital role in reducing soil pH, solubilizing Pi, and chelating P from iron and aluminum oxides (Khan et al., 2009). And bioavailability of P and soil P-nutrition greatly enhanced by inoculating PSB combined with application of organic manures (Adnan et al., 2017). Further, applying Silicon (Si) fertilizers (Na_2_SiO_3_) stimulates the wheat root exudation and changed the soil pH, as a result improved the Pi availability in rhizosphere; subsequently, upon Si-fertilization the upregulating expression of Pi transporters (*TaPHT1.1* and *TaPHT1.2*) significantly increases the P uptake (Kostic et al., 2017).

The organic combination of P management strategies and modern cultivation practices is equally important to treasure the soil resources and maintain plant health under adverse environmental conditions. Enhanced P management does not mean more P fertilizer application but efficient utilization of mineral resources with regard to the potential of agricultural productivity and ecological stability (Alewell et al., 2020).

## Conclusion and Prospects

Low temperature is one of the major abiotic factors that limits the wheat productivity under global climate change. In this review, we summarized the damaging impacts of LTS to wheat source–sink organs with respect to morphological, physiological, and molecular attributes (Tables 2, 3). Recently, under the continuous efforts of scientists around the world, great progress has been made in the research on the damaging mechanism of low temperature in wheat. LTS decreases the active leaf area and photosynthetic capacity, severely inhibits the expression of PS-related genes, resulting in reduced production of assimilates (Gan et al., 2019). LTS induced ABA pathways affect the sucrose metabolism and related gene expressions in spikelets, subsequently, sucrose transportation significantly diminished, thus leading to pollen abortion (Zhang et al., 2019).

As an essential plant nutrient, P can increase soil AP contents and improve the soil–plant nutrient and source–sink relationship, that resulted in enhanced LTS tolerance through reducing oxidative stress and increasing pollen activity. To enhance the PUE and cold tolerance of wheat, the crop husbandry practices, such as top dressing, mulched drip irrigation, deep application, and foliar spraying, should be opted according to different climatic zones (Li et al., 2019; Bindraban et al., 2020; Rafiullah et al., 2020). Further, it is needed to adopt conservation agricultural practices, especially application of P activators that promote the release of phosphate from soil particles (Zhu et al., 2018). Despite the ongoing developments in research field, some aspects still needs to be explored further and requires prospective studies, that is, stress sensing and signaling, molecular studies of the P regulation mechanism, the use of efficient breeding technologies, reducing gap between research and applied sectors through bridging research institutes and farmer communities (Zhang et al., 2021a). Hence, for better understanding of the stress responses, laboratory experiments should be combined with field experiments, and wheat plants should be focused than model plants (Dresselhaus and Huckelhoven, 2018; Hussain et al., 2018). Apart from this, breeding P-efficient and LTS-tolerant cultivars are also a pragmatic approach for future dealings with LTS and nutrient scarce soil conditions. Multidisciplinary cooperation is essential to build a platform for cooperative research concerning macro and micronutrient utilization and confronting abiotic stresses, thus mitigating the looming global P and food crisis.

## Author Contributions

HX and MAH conceived the concept of the review and prepared an outline of the review. HX, MAH, XC, DS, HF, and GJ compiled the literature and wrote different sections. BL, QN, WY, and ZW aided in designing figures and arranging references. XC and JL provided technical assistance and editing support. All authors contributed to the article and approved the submitted version.

## Funding

This work was supported by the Natural Science Foundation of Anhui Province (2008085QC122) and Major Science and Technology Projects in Anhui Province (202003b06020021).

## Conflict of Interest

The authors declare that the research was conducted in the absence of any commercial or financial relationships that could be construed as a potential conflict of interest.

## Publisher’s Note

All claims expressed in this article are solely those of the authors and do not necessarily represent those of their affiliated organizations, or those of the publisher, the editors and the reviewers. Any product that may be evaluated in this article, or claim that may be made by its manufacturer, is not guaranteed or endorsed by the publisher.
